# Extent of N-glycosylation of the metalloproteinase inhibitor and cytokine TIMP-1 determines pancreatic cancer cell proliferation and survival *via* CD63

**DOI:** 10.1016/j.jbc.2025.110211

**Published:** 2025-05-08

**Authors:** Daniel Häußler, Damjan Manevski, Julian Frädrich, Vanessa Brunner, Olga Prokopchuk, Alexander Sommer, Batu Toledo, Percy Knolle, Marc E. Martignoni, Helmut Friess, Paul Waterhouse, Achim Krüger

**Affiliations:** 1 TUM School of Medicine and Health, Institute of Experimental Oncology and Therapy Research, Technical University of Munich, Munich, Germany; 2 TUM School of Medicine and Health, Institute of Molecular Immunology, Technical University of Munich, Munich, Germany; 3 TUM School of Medicine and Health, Department of Surgery, Technical University of Munich, Munich, Germany; 4 Princess Margaret Cancer Centre, University Health Network, Department of Medical Biophysics, University of Toronto, Toronto, Canada

**Keywords:** TIMP-1, cytokine, protease inhibitor, multi-functional glycoproteins, glycosylation, glycosylation macroheterogeneity, pancreatic cancer, cancer-associated glycosylation changes, posttranslational modification

## Abstract

Glycosylation emerges as a critical determinant of protein function in cancer, yet its impact on multifunctional secreted factors remains understudied. Here, we identified tissue inhibitor of metalloproteinases-1 (TIMP-1), a glycoprotein with glycosylation sites at N30 and N78 harboring both canonical antiproteolytic and noncanonical cytokine-like activity, as one of the most upregulated secreted glycoproteins circulating in the blood of pancreatic cancer (PC) patients. Whereas plasma from healthy donors contained similar amounts of double-glycosylated (TIMP-1^glyc1/1^), single- glycosylated (N78 and not N30) (TIMP-1^glyc0/1^), and nonglycosylated (TIMP-1^glyc0/0^) TIMP-1, TIMP-1^glyc1/1^ predominated in plasma from PC patients. scRNAseq and *in vitro* validation linked this shift to tumor progression–associated upregulation of the oligosaccharyltransferase complex in epithelial cells. In human PC cell lines, oligosaccharyltransferase complex activity was critical for the synthesis of TIMP-1^glyc1/1^. Importantly, tumor cell survival and proliferation-promoting activity *via* CD63 were dependent on TIMP-1 glycosylation, which required N30-glycosylation. In contrast, glycosylation was not necessary for the antiproteolytic activity of TIMP-1 towards different matrix metalloproteinases (MMPs) (collagenases MMP-1, MMP-8; gelatinases MMP-2, MMP-9; stromelysin MMP-3; matrilysin MMP-7) but modulated the respective inhibitory efficacy. Analysis of a published glycoproteome data set, allowing assessment of individual glycosylation site occupancy in TIMP-1, revealed that N30 site occupation correlated with poor survival, while N78 site occupation showed no prognostic value, corroborating the impact of double glycosylation of TIMP-1, as observed in patients, on tumor promotion. The glycosylation-dependent modulation of the multifunctionality of tumor-secreted TIMP-1 thus provides a molecular basis for its long-debated cancer-promoting role. Finally, it exemplifies the impact of glycosylation macroheterogeneity on disease-relevant modulation of protein function.

In cancer, disease-associated posttranslational modifications (PTMs) of proteins can determine pathophysiological processes affecting intercellular communication (1), immune response (2), or tissue maintenance (3). Glycosylation of surface-associated and secreted proteins (4) represents the most stable and most common post-translational modification (5) emerging as a critical mechanism for cancer-induced alteration of protein function (2). Elucidation of the impact of the presence or absence of glycans on overall protein structure, and particularly the resulting function, emerges to be an important additional dimension for a better understanding of the role of individual genes in cancer (6). This level of phenotype regulation is necessary to expand our knowledge beyond the presence of a protein, as deduced from proteomic or even indirectly from transcriptomic analyses (7). While elucidation of the impact of presence or absence of glycans on monofunctional proteins has contributed to understanding their roles in a specific physiological (8) or pathophysiological (9) context, its consequence on the fine-tuning of the respective functions or prevalence of the respective activities of the many existing multifunctional glycoproteins (10) has so far not been addressed.

N-glycosylation is the enzymatic process by which glycans are covalently bound to the amide nitrogen of asparagine residues in proteins. This process starts in the endoplasmic reticulum (5). The transferred N-linked oligosaccharides contain multiple antennae, which undergo Golgi-mediated enzymatic modifications, generating diverse microheterogeneous glycoforms that modulate protein function (11). The extent of occupation of available N-glycosylation sites is primarily regulated by the endoplasmic reticulum–resident oligosaccharyltransferase (OST) complex, with STT3 as a key subunit (12). The STT3 protein exists as two isomers, STT3A and STT3B, and catalyzes the transfer of glycan-precursor structures to the acceptor sequence “asparagine-X-threonine/serine,” where X represents any amino acid except proline (13). Multiple studies have documented cancer-associated dysregulation of the OST complex expression (14, 15, 16), resulting in altered N-glycosylation site occupancy (17). Even though glycosylation macroheterogeneity was shown to determine protein function in principle (18), the functional consequences of cancer-associated changes in macroheterogeneity on surface and secreted proteins remained so far poorly understood. In particular, tumor-derived secreted glycoproteins, such as cytokines, are of high interest as cancer-dependent glycosylation changes of these proteins modulate the paracrine signaling mechanisms that emerge as important determinants shaping the local and systemic cancer-promoting environment (19). One important multifunctional secreted glycoprotein is tissue inhibitor of metalloproteinases 1 (TIMP-1) (20), which exhibits a canonical antiproteolytic activity towards metalloproteinases, including various matrix metalloproteinases (MMPs) (21), as well as a noncanonical cytokine-like function *via* several cell surface receptors (20, 22), including the interaction with the tetraspanin CD63, inducing increased proliferation (23) and survival of tumor cells (24) pointing at its tumor-promoting function in most cancers (25).

Pancreatic cancer (PC) is a highly aggressive tumor entity where protein variants with a wide variety of glycosylation patterns are expressed (26). In the present study, we focused on the PC-derived secretome of glycosylated proteins aiming at the identification of a multifunctional secreted glycoprotein. Here, we identified TIMP-1 as one of the most upregulated secreted glycoproteins in PC, which served as a model addressing the impact of glycosylation pattern on its multifunctionality. Functional analyses with PC cell lines unraveled a novel link between glycosylation macroheterogeneity and the multifunctionality of TIMP-1.

## Results

### TIMP-1 glycosylation variants in the plasma of pancreatic ductal adenocarcinoma patients

**Figure 1:**
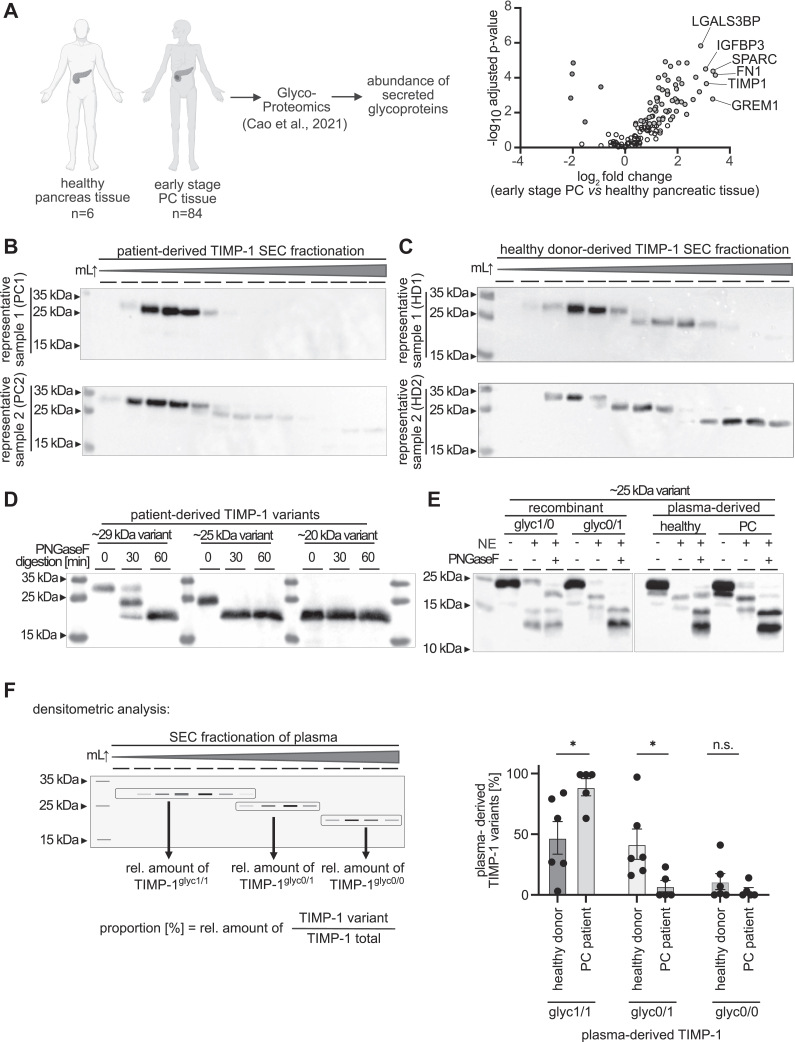
**TIMP-1 glycosylation macroheterogeneity is altered in the plasma of PC patients.***A*, volcano plot comparing the abundance of glycoproteins in normal pancreas tissue and tissue from early-stage pancreatic cancers. *B*/*C*, representative western blots of purified TIMP-1 in sequential fractions after size-exclusion chromatography (SEC) of plasma from pancreatic cancer patients (*B*) and healthy individuals (*C*). *D*, Western blot of 0, 30, and 60 min PNGase F digest of TIMP-1 in selected SEC fractions of plasma from a PC patient (PC5, see Fig. S1*A*). *E*, TIMP-1 Western blot of the neutrophil elastase and PNGase F digest of monoglycosylated recombinant TIMP-1 proteins and of monoglycosylated TIMP-1 SEC plasma fractions from a healthy individual (HD2) and a pancreatic patient (PC5). *F*, quantification of the band intensity for double, mono, and nonglycosylated TIMP-1 detected in the TIMP-1 western blots of fractionated plasma from healthy individuals (n = 6) and pancreatic cancer patients (n = 5). Also see Fig. S1*A*, *B*, and *D*. Statistical analysis were performed by Student’s t-tests or Mann Whitney tests (*F*). Mean ± SD (*F*) ∗, *p* ≤ 0.05.

We analyzed a glycoproteome dataset of early-stage pancreatic tumors and healthy pancreatic tissue samples (27) to identify differentially abundant secreted glycoproteins. Using stringent filtering criteria (adjusted *p*-value < 0.05), we performed differential abundance analysis to identify glycoproteins upregulated in early-stage PC tissue compared to healthy tissue. From this analysis, we sought to identify multifunctional candidates with potential functional significance in PC progression. The analysis lead to the identification of TIMP-1, FN-1, SPARC, IGFBP-3, LGALS3BP, and GREM1 (Fig. 1*A*) as the most upregulated glycoproteins in early-stage pancreatic tumor tissues as compared to controls. Of these glycoproteins, we selected TIMP-1 as a candidate for investigating glycosylation-mediated functional modulation due to its previously described multifunctionality (20). Multifunctionality of TIMP-1 comprises its antiproteolytic functions (inhibition of disease progression-promoting MMPs (28)) and its cancer-promoting cytokine-like (29) activity *via* interactions with cell surface receptors (20, 25, 30). In an orientation study with few healthy donors (n = 6) and treatment-naïve patients (n = 5), who could donate the necessary large amount of blood (35 ml) for us to purify detectable amounts of TIMP-1 protein (see Experimental procedures), we aimed to assess the potential existence and spectrum of differential macroheterogenous glycosylation of the secreted TIMP-1 present in PC patients or healthy donors. We purified protein from their blood plasma (Figs. 1*B* and S1*A*) (Figs. 1*C* and S1*B*), employing an improved (31) high-capacity size-exclusion chromatography (SEC)-based fast protein liquid chromatography protocol to fractionate all possible glycosylation variants of TIMP-1 (Fig. S1*C*). Western blot analysis of purified TIMP-1 fractions revealed three distinct bands at ∼29 kDa, ∼25 kDa, and ∼20 kDa, suggesting three TIMP-1 glycosylation variants. To confirm that these three bands reflect differential glycosylation, we used PNGaseF to deglycosylate SEC fractions containing each of the TIMP-1 variants (Fig. 1*D*). We observed that the ∼29 kDa band corresponded to double-glycosylated TIMP-1 (dgTIMP-1), the ∼25 kDa band to monoglycosylated TIMP-1, and the ∼20 kDa band to nonglycosylated TIMP-1, revealing a patient-specific distinct pattern of a majority of dgTIMP-1 in PC patients as compared to a rather equal distribution of all variants in healthy donors. Given that TIMP-1 contains two glycosylation sites (N30 and N78) (25, 28), it was necessary to determine whether the monoglycosylated TIMP-1 variant-containing band at ∼25 kDa represents a mixture of TIMP-1 glycosylated at either N30 or N78 or if only one of these sites is preferably occupied. To this end, we digested the SEC fractions containing the ∼25 kDa variant from PC patients and healthy controls with neutrophil elastase, which cleaves TIMP-1 between the glycosylation sites (Fig. S1*D*), yielding up to three antibody-detectable peptides (undigested ∼25 kDa peptide, partial digested ∼18 kDa peptide, full digested ∼16 kDa peptide), that all harbor the N78 glycosylation site. These peptides were subsequently deglycosylated with PNGaseF (Fig. 1*E*). Comparison of the digestion patterns between recombinant TIMP-1 (rTIMP-1) variants harboring glycans at either N30 or N78 (Fig. S1, *D*–*F*) and plasma-derived TIMP-1 revealed that the monoglycosylated plasma-derived TIMP-1 variant is glycosylated at N78, with no detectable variant glycosylated at N30. Densitometric analysis was employed to quantify the relative abundance of TIMP-1 variants in plasma from PC patients and healthy controls (Fig. 1*F*). We observed a shift towards increased abundance of dgTIMP-1 in PC patient plasma as compared to healthy donors–derived plasma. Altogether, these analyses identified the existence of only three of the theoretically possible four distinct TIMP-1 glycosylation variants in healthy donors and PC patients: the fully glycosylated form (TIMP-1^glyc1/1^), only one of the two possible monoglycosylated forms glycosylated at N78 (TIMP-1^glyc0/1^), and the nonglycosylated form (TIMP-1^glyc0/0^). The enrichment of plasma-derived TIMP-1^glyc1/1^ in PC suggests a cancer-associated alteration in TIMP-1 glycosylation macroheterogeneity.

### PC-associated increase in OST complex expression alters TIMP-1 glycosylation macroheterogeneity

**Figure 2:**
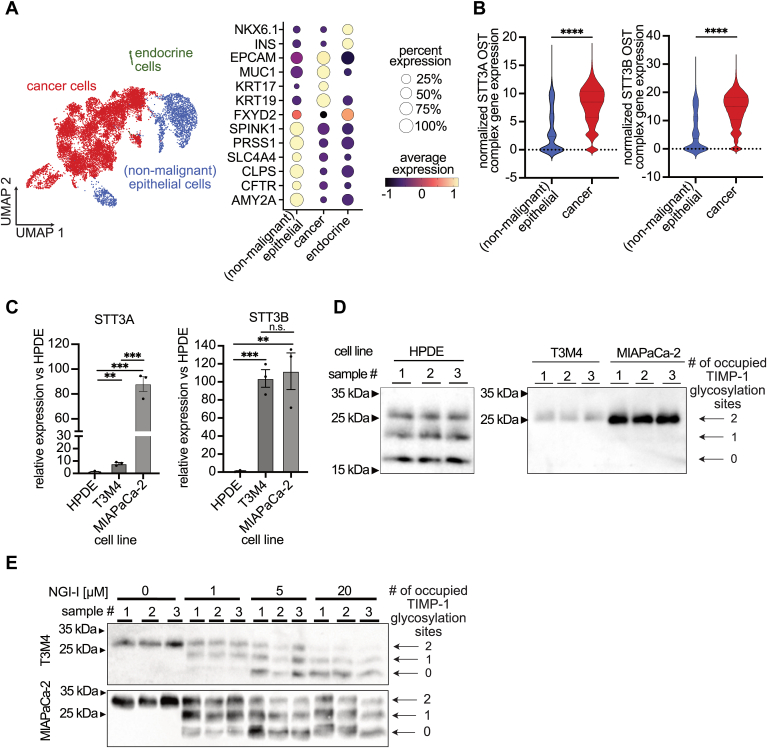
**PC-associated increase in oligosaccharyltransferase complex expression alters TIMP-1 glycosylation macroheterogeneity.***A*, UMAP of epithelial single cells (selected from all cells) of samples from pancreatic tumor tissues (n = 19) from the Steele dataset. Populations are identified by color (see legend). Dot plot of key markers used to define the identified cell populations. The color of each dot represents the average expression, while the size of the dot represents the percentage expression in the respective cell population. The gene expression of lineage markers have been merged for all samples. *B*, violin plot of the average sum expression of STT3A and STT3B OST complex of each cell clusters. *C*, relative STT3A and STT3B expression in the nontumorous pancreatic cell line HPDE and the pancreatic tumor cell lines MIAPaCa-2 and T3M4. *D*, TIMP-1 western blots of the supernatant of nontumorous pancreatic HPDE cells and pancreatic tumor cell lines MIAPaCa-2 and T3M4. The number of occupied TIMP-1 glycosylation sites is indicated by arrows (two occupied sites corresponds to ∼28.5 kDa, one occupied site to ∼24 kDa, and no glycosylation corresponds to 22.7 kDa). Sample number reflects three biological replicates. *E*, TIMP-1 western blots of the supernatant of pancreatic cancer cell lines MIAPaCa-2 and T3M4 after treatment with 0, 1, 5, or 20 μM NGI-1 for 48h. MW of glycosylation variants as in (*D*). Sample number reflects three biological replicates Statistical analysis were performed by Student’s t-tests or Mann Whitney tests (*B* and *C*). Mean ± SD (*B*) ∗∗*p* ≤ 0.01; ∗∗∗*p* ≤ 0.001; ∗∗∗∗*p* ≤ 0.0001.

After identification of altered patterns of glycosylated TIMP-1 variants from a more even distribution of TIMP-1^glyc1/1^, TIMP-1^glyc0/1^, and TIMP-1^glyc0/0^ in the plasma of healthy donors to a strong prevalence of TIMP-1^glyc1/1^ in PC patient plasma (Fig. 1), we next addressed the underlying molecular mechanism. Since OST complex is decisive for efficient N-glycosylation site occupancy (8) and known to be induced in many cancer types other than PC, we analyzed single-cell RNA-seq data from PC (32) and annotated them with published markers (32, 33) in order to distinguish the clusters of PC cells from nonmalignant epithelial cells (Fig. 2*A*). PC cells exhibited increased expression of both STT3A and STT3B isomers, as well as other OST complex subunits (Figs. 2*B* and S2*A*). Consistent with this finding, PC cell lines T3M4 and MIAPaCa-2 exhibited higher expression levels of STT3A and STT3B than the nontumorous pancreas cell line HPDE (Fig. 2*C*). We also demonstrated a shift from a mixture of non-, partially-, and fully- glycosylated to essentially only fully glycosylated secreted TIMP-1 in supernatants, which correlated with increased STT3A and STT3B expression in these cell lines (Figs. 2*D* and S2*B*). Treatment of T3M4 and MIAPaCa-2 cells with the OST glycosyltransferase inhibitor NGI-1 altered the proportions of TIMP-1 variants, reducing the amount of TIMP-1^glyc1/1^, while increasing the amounts of TIMP-1^glyc0/1^ and TIMP-1^glyc0/0^ variants in the cell culture supernatant (Figs. 2*E* and S2*C*). Taken together, these results suggest that alterations in OST complex expression in PC cells are mirrored in the shift of the respective glycosylation variants of TIMP-1 observed in the plasma of PC patients compared to healthy donors (Fig. 1).

### The tumor-promoting cytokine-like activity of TIMP-1 is modulated by glycosylation macroheterogeneity

**Figure 3:**
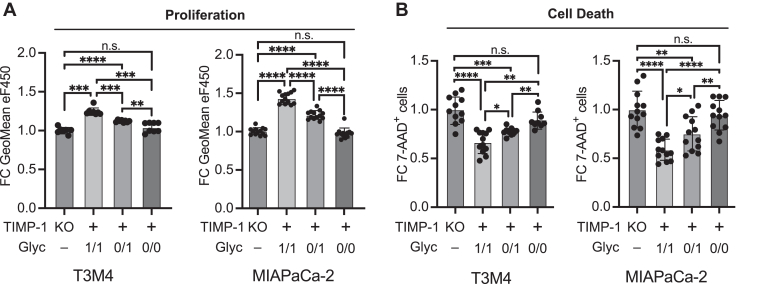
**TIMP-1 glycosylation macroheterogeneity determines the tumor-promoting cytokine-like activity in pancreatic cancer cells.***A*, quantification of proliferation by flow cytometry. T3M4^T1KO^ or MIAPaCa-2^T1KO^ cells expressing transgenic TIMP-1^glyc1/1^, TIMP-1^glyc0/1^, or TIMP-1^glyc0/0^ or control (T1KO) cells were stained with eFluor450 proliferation dye and cultured for 48h. Proliferation was evaluated by calculation of the geometric means of eFluor450 staining. The background of non-eFluor450–stained cells was subtracted, and fold change of the reciprocal eFluor450 geometric means was calculated by normalization to control cells. Data represents n = 8 for T3M4^T1KO^ and n = 13 biological replicates for MIAPaCa-2^T1KO^. *B*, quantification of cell death by flow cytometry. T3M4^T1KO^ or MIAPaCa-2^T1KO^ cells expressing either TIMP-1^glyc1/1^, TIMP-1^glyc0/1^, or TIMP-1^glyc0/0^ or control TIMP-1^ko^ cells were cultivated for 48h and amount of death cells were analyzed by positive staining for 7-AAD. Fold change of percentages was calculated by normalization to control cells. Data represents n = 12 biological replicates for T3M4^T1KO^ and MIAPaCa-2^T1KO^ experiments. TIMP-1^glyc1/1^ harbors two glycans at N30 and N78, TIMP-1^glyc0/1^ harbors one glycan at N78 and no glycan N30, and TIMP-1^glyc0/0^ harbors no glycans at N30 and N78. Statistical analysis was performed by Student’s t-tests or Mann Whitney tests (*A* and *B*), Mean ± SD (*A* and *B*) ∗*p* ≤ 0.05; ∗∗*p* ≤ 0.01; ∗∗∗*p* ≤ 0.001; ∗∗∗∗*p* ≤ 0.0001.

**Figure 4:**
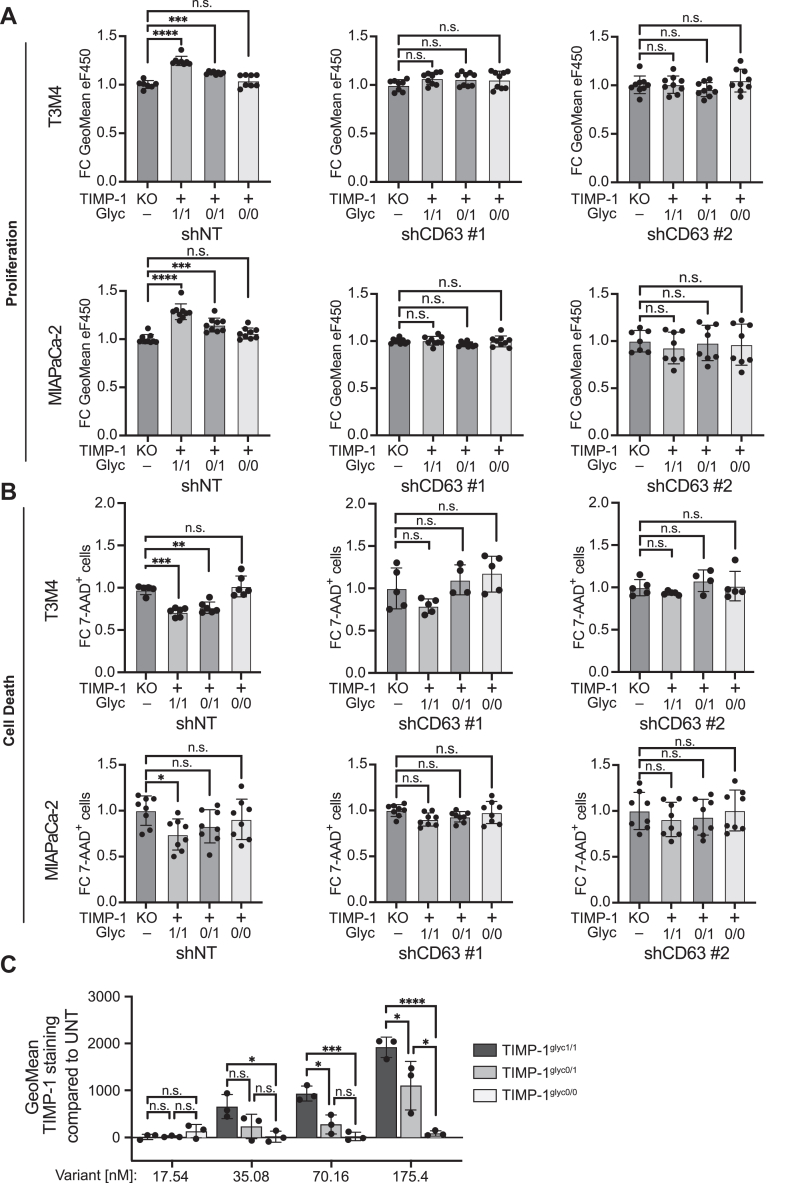
**Glycosylation status of TIMP-1 determines its CD63-dependent tumor-promoting cytokine-like activity in pancreatic cancer cells.***A*, quantification of proliferation by flow cytometry of eFluor450-stained cell lines. T3M4 and MIAPaCa-2 cells expressing TIMP-1 glycosylation variants glyc1/1, glyc0/1, glyc0/0, or control TIMP-1^ko^ cells with (shNT) and without (shCD63#1 and shCD63#2) CD63 knockdown were stained with eFluor450 proliferation dye and cultivated for 48h. Data represents n = 9 biological replicates for T3M4^T1KO^ or MIAPaCa-2^T1KO^. *B*, quantification of cell death by flow cytometry. T3M4 and MIAPaCa-2 TIMP-1 glycosylation variants or control cells (T1KO) with (shNT) and without (shCD63#1 and shCD63#2) CD63 knockdown were cultured for 48h and cell death was determined by positive staining for 7-AAD. Fold change of percentages were calculated by normalization to control cells. Data represents n = 6 biological replicates for T3M4^T1KO^ experiments, n = 9 biological replicates for MIAPaCa-2^T1KO^ experiments. *C*, flow cytometry–based TIMP-1/CD63–binding assay. T3M4^T1KO^ cells were incubated for 10 min with rTIMP-1^glyc1/1^, rTIMP-1^glyc0/1^, or rTIMP-1^glyc0/0^ and binding of TIMP-1 to CD63 was analyzed by calculation of the geometric means of TIMP-1 staining on CD63-positive cells. TIMP-1^glyc1/1^ harbors two glycans at N30 and N78, TIMP-1^glyc0/1^ harbors one glycan at N78 and no glycan N30, and TIMP-1^glyc0/0^ harbors no glycans at N30 and N78. Statistical analysis was performed by one-way ANOVA using Dunnett's multiple comparisons test comparing multiple groups against the control cells (*A* and *B*) or Student’s t-tests or Mann Whitney tests (*C*), Mean ± SD (*A*–*C*) ∗*p* ≤ 0.05; ∗∗*p* ≤ 0.01; ∗∗∗*p* ≤ 0.001; ∗∗∗∗*p* ≤ 0.0001.

Next, we functionally addressed the question, whether a shift to fully glycosylated TIMP-1^glyc1/1^ can be linked to noncanonic CD63-mediated tumor-promoting effects of TIMP-1, namely tumor cell proliferation and survival (23, 24). To this end, we first generated TIMP-1–ablated PC cell models with T3M4 and MIAPaCa-2 cell lines (Fig. S3, *A*–*D*) in which we could determine the function of differentially glycosylated TIMP-1 variants on these parameters without the interference of endogenously expressed TIMP-1 (Fig. 2*D*). Of note, proliferation and survival of TIMP-1–expressing T3M4 and MIAPaCa-2 cell lines (Fig. 2*D*) were reduced upon TIMP-1 ablation (Fig. S3*E*). We then genetically engineered the TIMP-1 KO cell lines to express only one of the TIMP-1 glycosylation variants (Fig. S3, *F* and *G*). Overexpression and secretion of TIMP-1^glyc 0/0^ did not show any proliferation or survival-promoting effect (Fig. 3, *A* and *B*) in T3M4^T1KO^ and MIAPaCa-2^T1KO^ cell lines as compared to controls. T3M4^T1KO^ and MIAPaCa-2^T1KO^ cell lines overexpressing and secreting TIMP-1^glyc 1/1^ or TIMP-1^glyc 0/1^ showed enhanced proliferation (Fig. 3*A*) and survival (Fig. 3*B*) compared to controls. Notably, secreted TIMP-1^glyc 1/1^ harboring both glycans at N30 and N78 was more potent than the TIMP-1^glyc 0/1^ variant harboring the glycan at N78 alone, demonstrating that the occupation of the N30 glycosylation site, as observed in PC patient plasma (Fig. 1), is decisive for TIMP-1 to exhibit its full tumor cell-promoting cytokine potential. Knockdown of the TIMP-1 receptor CD63 in these cells (Fig. S3*H*) abolished the promoting effects of secreted TIMP-1^glyc 1/1^ and TIMP-1^glyc 0/1^ variants on proliferation (Fig. 4*A*) and survival (Fig. 4*B*). Next, we employed a flow cytometry–based binding assay with escalating doses of the recombinant TIMP-1 variants rTIMP-1^glyc 1/1^, rTIMP-1^glyc 0/1^, and rTIMP-1^glyc 0/0^ added to T3M4^T1KO^ cells (Fig. S1, *E*–*G*), revealing glycosylation dependency of TIMP-1/CD63–binding efficacy on cells (Fig. 4*C*). This incremental binding efficacy correlated with the strength of the effect of the glycosylation variants on tumor cell proliferation (Figs. 3*A* and 4*A*) and survival (Figs. 3*B* and 4*B*), corroborating that the tumor-promoting cytokine-like effect of differentially glycosylated TIMP-1 was mediated by its interaction with CD63.

### Glycosylation macroheterogeneity fine-tunes the antiproteolytic function of TIMP-1

**Figure 5:**
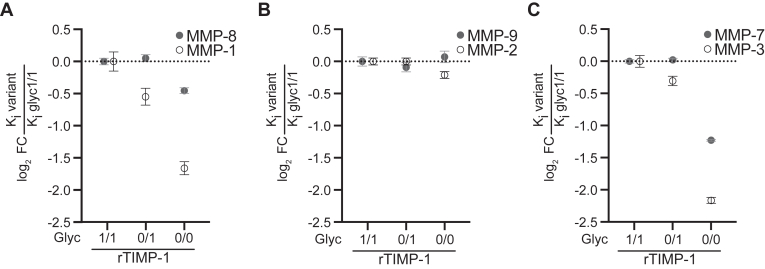
**Glycosylation macroheterogeneity modulates the antiproteolytic function of TIMP-1.** The inhibitory capacity of TIMP-1 against MMPs is modulated by its glycosylation macroheterogeneity. *A*–*C*, comparison of K_i_ values of the different TIMP-1 glycosylation variants for different MMPs classes: collagenases (*A*), gelatinases (*B*), stromelysin (*C*), and matrilysin (*C*). Fold change was calculated by dividing the K_i_ of TIMP-1^glyc1/1^ by the K_i_ of the different TIMP-1 glycosylation variants for the respective MMPs. Mean ± SD.

So far, our results identified glycosylation macroheterogeneity as a critical determinant of the tumor-promoting cytokine-like activity of TIMP-1 *via* CD63 (Figs. 3 and 4), which would be one explanation for the correlation of elevated expression of double-glycosylated TIMP-1 in PC. Although the antiproteolytic activity of TIMP-1 does not seem to be effective in tumor inhibition (20, 25), we still wanted to address whether glycosylation macroheterogeneity affects this canonical function of TIMP-1 on selected classes of MMPs (collagenases, gelatinases, stromelysin, matrilysin). However, the analysis of TIMP-1 glycosylation-dependent effects on each MMP in a cellular system would be inconclusive since MMPs are known to influence each other’s activity in a cellular context (34). Therefore, we tested the antiproteolytic activity of the glycosylation variants of TIMP-1 in biochemical assays employing recombinant human MMPs and TIMP-1 glycosylation variants. The respective recombinant MMPs were incubated with increasing concentrations of the recombinant TIMP-1 variants rTIMP-1^glyc^^1/1^, rTIMP-1^glyc^^0/1^, and rTIMP-1^glyc^^0/0^ (Fig. S1, *E*–*G*) and cleavage of fluorogenic substrates FS-6 or NFF-1 determined. Based on respective MMP activity/TIMP-1 concentration curves, the inhibitory constant (K_i_) and 50% inhibitory concentration (IC_50_) were calculated. Glycosylation of TIMP-1 did not affect the inhibition of gelatinases MMP-2 and MMP-9 (Table 1 and Fig. 5*B*). In contrast, different glycosylation variants of TIMP-1 yielded in differential inhibition of the tested collagenases (MMPs-1, -8), stromelysin (MMP-3), and matrilysin (MMP-7) (Table 1 and Fig. 5, *A* and *C*), revealing a modulation of the antiproteolytic function. We observed an increase in the inhibition of MMPs with reduced TIMP-1 glycosylation status for MMP-1 (Fig. 5*A*) and MMP-3 (Fig. 5*C*) with rTIMP-1^glyc^^0/0^ as the most potent inhibitor. Similarly, MMP-7 (Fig. 5*C*) and MMP-8 (Fig. 5*A*) were most potently inhibited by rTIMP-1^glyc^^0/0^, whereas rTIMP-1^glyc^^1/1^ and rTIMP-1^glyc^^0/1^ were less potent inhibitors. Taken together, we observe that as TIMP-1 glycosylation decreases, its antiproteolytic activity against MMPs progressively increases. Specifically, rTIMP-1^glyc0/1^ shows increased inhibition of MMP-1 and MMP-3, while rTIMP-1^glyc^^0/0^ shows increased inhibition of all investigated MMPs except gelatinases. This demonstrates that TIMP-1 glycosylation is not necessary for the execution but rather for the modulation of the antiproteolytic activity of TIMP-1, while glycosylation was necessary to execute the tumor-promoting cytokine-like effects of TIMP-1 *via* CD63 (Figs. 3 and 4).

**Table 1:** K_i_ and IC_50_ values of TIMP-1 glycosylation variants with MMPs

K_i_±SD [pM]				
MMP-class	MMP	rTIMP-1		
		Glyc1/1	Glyc0/1	Glyc0/0
Collagenases	MMP-1	20.55 ± 2.01	14.06 ± 1.76	6.50 ± 1.36∗
	MMP-8	34.98 ± 1.13	36.26 ± 1.19	25.55 ± 0.95∗
Gelatinases	MMP-2	370.3 ± 15.3	373-0 ± 16.0	318.4 ± 95.9
	MMP-9	991.0 ± 62.0	929.3 ± 59.8	1041.2 ± 74.1
Stromelysin	MMP-3	78.70 ± 5.75	63.75 ± 4.60	17.54 ± 2.94∗
Matrilysin	MMP-7	3.09 ± 0.07	3.14 ± 0.08	1.32 ± 0.04∗


IC_50_±SD [nM]				
MMP-class	MMP	rTIMP-1		
		Glyc1/1	Glyc0/1	Glyc0/0
Collagenases	MMP-1	0.35 ± 0.06	0.28 ± 0.04	0.17 ± 0.02∗
	MMP-8	1.34 ± 0.08	1.47 ± 0.09	1.03 ± 0.05∗
Gelatinases	MMP-2	0.65 ± 0.08	0.71 ± 0.14	0.93 ± 0.12
	MMP-9	2.38 ± 0.33	1.85 ± 0.34	2.20 ± 0.47
Stromelysin	MMP-3	0.91 ± 0.08	0.7 ± 0.04∗	0.49 ± 0.03∗
Matrilysin	MMP-7	0.21 ± 0.01	0.21 ± 0.01	0.12 ± 0.01∗

Calculated inhibitory constants (K_i_) (upper panel) and IC_50_ (lower panel) of the recombinant TIMP-1 glycosylation variants with the full-length human MMPs. K_i_ values were estimated using the Morrison equation for tight binding inhibitors, and the IC_50_- values were estimated using a four-variable inhibitor concentration *versus* response equation. Statistical analysis was performed by extra sum-of-squares F test based on the best-fit values. Mean ± SD. ∗*p* ≤ 0.05.

### Correlation of PC progression with occupation of the N30 glycosylation site of TIMP-1

**Figure 6:**
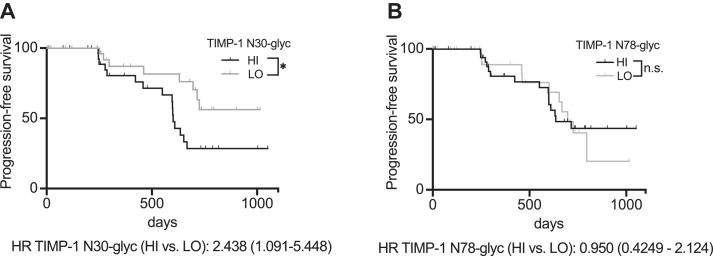
**PC patients with increased TIMP-1 N30 glycosylation levels show shortened progression-free survival.***A*, progression-free survival of PC patients (M0, R0) with high glycosylation site occupation at position N30 (TIMP-1 N30-glyc^HI^) in the pancreatic primary tumor as to patients with low glycosylation site occupation at position N30 (TIMP-1 N30-glyc^LO^). *B*, probability of progression-free survival of PC patients (M0, R0) with high glycosylation site occupation at position N78 (TIMP-1 N78-glyc^HI^) *versus* patients with low glycosylation site occupation at position N78 (TIMP-1 N78-glyc^LO^). The log-rank test was employed for statistics. ∗*p* ≤ 0.05.

Our previous experiments demonstrated that glycosylation macroheterogeneity of TIMP-1 modulates its multifunctionality, where N30-glycosylation was important for the tumor-promoting cytokine-like activity. This raised the question whether differential occupation of N30 and N78 can be demonstrated to be important for cancer survival, in addition to the shift observed in the orientation study (Fig. 1). We addressed this question using the glycoproteome data set of PC patients (27). Of note, while this data set allowed us to quantify N30 and N78 glycosylation site occupation of TIMP-1, it could not distinguish between the specific native TIMP-1 glycoforms that we had observed in plasma of PC patients and healthy donors (Fig. 1). This analysis revealed a clear correlation between N30 glycosylation site occupation and shortened survival (Fig. 6*A*), while occupation of N78 did not correlate with survival (Fig. 6*B*). In particular, high levels of N30 glycosylation site occupation (TIMP-1 N30glyc^HI^) was identified as an indicator of an increased mortality risk of PC patients, corroborating the impact of N30 glycosylation site occupation of TIMP-1 on cancer progression.

## Discussion

This study establishes the impact of differential glycosylation on the disease (cancer)-associated multifunctionality of TIMP-1. While glycosylation was not necessary for the overall antiproteolytic activity of TIMP-1, we here demonstrate an impact of glycosylation on this function, namely that differential glycosylation site occupancy modulated the selective antiproteolytic capacity towards different MMP classes. This may explain why studies employing recombinant TIMP-1 from bacterial source (35, 36, 37) tended to emphasize the antiproteolytic function of TIMP-1, while studies employing glycosylated recombinant TIMP-1 (38, 39) led to the (re-) discovery of its protumorigenic signaling *via* the cell surface tetraspanin CD63 (24, 40) or cytokine-like activity (22, 29, 41), respectively. The multifunctional ability of TIMP-1 to inhibit tumor-promoting metalloproteinases on the one hand and to trigger protumorigenic signals on the other hand, representing a paradox in cancer (42), initiated questions concerning the structure-function relationship of TIMP-1 (20, 25, 29). Notably, in patients, clear correlations between elevated blood levels of TIMP-1 and progression of virtually all inflammatory diseases, including cancer, have been described (43, 44, 45). Towards elucidation of structure-function relationships within the TIMP-1 protein, the canonical protease inhibitory activity was assigned with a conserved CTC motif in the N-terminal domain (21), and the noncanonical signaling function was shown to be dependent on the presence of the last nine amino acids (WGSLRSQIA) of the C-terminal domain (46) or a moonlighting function involving the N-terminal domain (41). In the present study, we now included the PTM of glycosylation into these considerations. In fact, we could demonstrate here that changes in glycosylation site occupancy differentially impact on both functions. Even more, we could link structure-function relationships based on glycosylation occupancy to the manifestation of PC in patients. In specific, while glycosylation at N78 is essential for the cytokine activity of TIMP-1, we demonstrate that the additional occupation at N30 led to full exhibition of its cytokine effect.

In the present study, we found evidence that glycosylation shifts favor the noncanonical disease-promoting function of TIMP-1 in PC. This notion was deduced from our initial observations, subsequent analysis of structure-function relationships, and survival analysis of available glycoproteome data from PC patients. Firstly, the monoglycosylated form of TIMP-1, as detected in the plasma of healthy donors and PC patients, consisted of N78-glycosylated TIMP-1, and N30 was only occupied in the double-glycosylated form. This does not totally exclude the possibility of local expression and secretion of an N30-occupied monoglycosylated variant in the tumor, but this variant did not become apparent in the plasma. Secondly, double glycosylated TIMP-1 was the most efficient TIMP-1 variant to interact with CD63, promoting tumor cell proliferation and survival. Thirdly and finally, increased N30 glycosylation site occupation of TIMP-1 correlated with shortened survival of PC patients. Altogether, TIMP-1 variants with different functional abilities coexist in the healthy donor which may be involved in the maintenance of tissue homeostasis *via* the antiproteolytic activity of TIMP-1 and regulation of immune cell function *via* the cytokine-like activity of TIMP-1, whereas the shift to the latter signifies the transition into pathophysiological conditions. Our findings explain a previous study demonstrating that only double-glycosylated TIMP-1 but not nonglycosylated TIMP-1 was able to increase tumor growth *in vivo* (47). Here, we show that disease-associated double glycosylation of TIMP-1 might causally and mechanistically be related to the cancer-associated increase of the OST complex expression and activity, typical for cancer progression (14, 15, 48).

**Figure 7:**
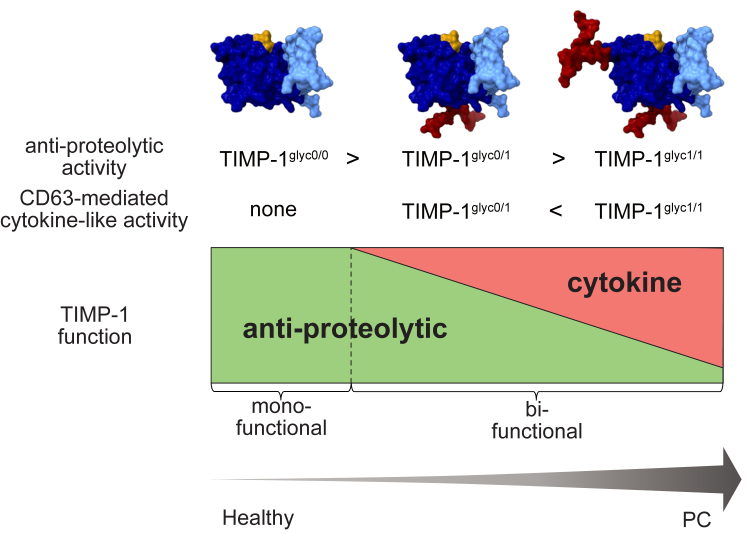
**TIMP-1 glycosylation macroheterogeneity modulates its multifunctionality.** Graphical depiction of the modulation of TIMP-1s multifunctionality by its glycosylation macroheterogeneity identified in the plasma of humans. For the antiproteolytic activity, TIMP-1^glyc0/0^ exhibited the strongest inhibitory capacity, followed by TIMP-1^glyc0/1^ and TIMP-1^glyc1/1^, which showed the lowest inhibitory capacity (see Fig. 5 and Tab. 1). Regarding the CD63-mediated tumor-promoting cytokine-like activity, TIMP-1^glyc1/1^ induced the strongest cytokine-like activity, measured by proliferation and survival assays, followed by TIMP-1^glyc0/1^. No cytokine-like activity was induced by TIMP-1^glyc0/0^. Based on this, TIMP-1^glyc0/0^ is a monofunctional protein, whereas TIMP-1^glyc0/1^ and TIMP-1^glyc1/1^ are multifunctional proteins. The shift towards increased TIMP-1 glycosylation site occupation correlates with PC disease progression and increased cytokine activity. TIMP-1 structures were generated using the modeled using AlphaFold2 and representative glycans from the Glycam webserver. Color coding of the TIMP-1 structures: Glycans: *red*; N-terminal domain: *dark blue*; C-terminal domain: *light blue*; anti-proteolytic CTC-motif: *orange*.

Here, as exemplified with TIMP-1, we shed light on the notion that glycosylation macroheterogeneity can allow molecular incremental modulation of one function of the protein, in the case of TIMP-1, the anti-proteolytic activity, to the other function of the protein, in the case of TIMP-1, the cytokine-like activity (Fig. 7). This is a significant expansion to the previous studies addressing the impact of glycosylation site occupancy, as exemplified with integrin α5β1, on the activity of either ligand binding (49) on the one hand or the activation of intracellular signaling (50) on the other hand. These separate functions were each dependent on the presence or absence of glycosylation (49, 50, 51), while we show an incremental modulation between two biologically opposing functionalities (antiproteolytic and cytokine-like) within one multifunctional protein (Fig. 7). Furthermore, we here were able to relate these biochemical and functional findings—observed and tested with human proteins and human cell lines—to the clinical situation of pancreas cancer, where glycoproteomic data, survival data, and as a first orientation study with fresh blood samples from treatment-naïve patients, were available. We found this important, because glycosylation patterns are known to vary significantly between humans and rodents (52, 53), which can influence the functional outcomes of studies focusing on glycosylation (54).

Finally, elucidating the impact of glycosylation may not only resolve different or even opposing views on the functions of individual proteins, for example, the long-debated TIMP-1 paradox in cancer (42), but also offers new opportunities for clinical diagnostics and therapeutic intervention targeting cancer-associated protein glycoforms.

## Experimental procedures

### Patients and clinical data

The orientation study included samples and clinical data from patients diagnosed with PC, who underwent oncological treatment between 2020 and 2023 in the Department of Surgery, TUM School of Medicine and Health, Technical University of Munich. The study reported in this manuscript were conducted in accordance with the ethical principles outlined in the Declaration of Helsinki and approved by the Ethics Committee of the Medical Faculty (TUM School of Medicine and Health) of the Technical University of Munich (Germany; #409/16S, ‘*Deutsches Register Klinischer Studien*’ DRKS00017285; #552/18S). Written informed consent was obtained from all participants and all individuals agreed to participate before inclusion in the study. The analysis was conducted on a pseudonymized data set. Diagnosis of PC was verified by definitive histological examination of surgical specimens or retrieved biopsies using the eighth edition of the UICC tumor, node, metastasis classification, and staging system for PC (55). In this study, we recruited only five treatment-naïve PC patients due to strict inclusion criteria. Inclusion in the study required PC patients to be treatment-naïve and to consent to donating 35 ml of blood shortly before surgery. These inclusion criteria limited the eligible number of patients for study recruitment for several reasons: some patients were unable to donate blood in addition to routine clinical blood draws, anesthesiology teams excluded patients to minimize preoperative blood loss, and the increasing prevalence of neoadjuvant therapy in modern treatment protocols (56) rendered many patients ineligible. Blood was collected in a 2.7-ml EDTA-coated tube (S-Monovette; Sarstedt) and mixed immediately by gently inverting the tube after collection. Blood plasma was obtained within 30 min by centrifugation of whole blood for 15 min at 1000*g*. Plasma samples were immediately snap frozen in liquid nitrogen and stored at −80 °C.

### Cell lines

Human MIAPaCa-2 PC cell line was obtained from ATCC (cat#: CRL-1420) and human and T3M4 PC cell line obtained from AcceGen (cat#:ABC-TC1324). Both cell lines were cultured in Dulbecco’s modified Eagle’s medium (DMEM, Pan Biotech GmbH, Ort) supplemented with 10% fetal bovine serum (FBS) and 1% glutamine at 37°C, 5% CO_2_. The human pancreatic epithelial cell line HPDE was retrieved from Kerafast inc (cat#: ECA001-FP). HPDE cells were cultured at 37 °C, 5% CO_2_ in keratinocyte SFM media (Gibco) supplemented with human recombinant epidermal growth factor and bovine pituitary extract as provided by the manufacturer.

### Analysis of glycoproteome data from human samples

The abundance of glycoproteins in early-stage pancreatic ductal adenocarcinoma *versus* normal pancreas tissue was analyzed using a published glycoproteomic data set (27) (CPTAC data portal accession numbers PDC000272 and PDC000270). For analysis of most upregulated glycoproteins in early-stage tumors versus normal pancreas tissues, log_2_FC and adjusted *p*-values were retrieved from (27) (′DIA_EarlyStagevsNormalDuct_log_2_FC′ and `DIA_EarlyStagevsNormalDuct_adjusted_p′ from Cao *et al.*, ′Glycoprotein_earlystage_kras′). Glycoproteins were filtered for secreted glycoproteins employing the cellular location annotation ′Secreted(blood)′ (′Cellular_Location` from Cao *et al.*, ′Glycoprotein_Tumor_vs_NAT`). Fold change and adjusted *p*-value of the abundance of all secreted glycoproteins comparing pancreatic tumors versus normal pancreas duct were depicted in a volcano plot. For analysis of TIMP-1 glycosylation site occupation in PC patients, the published data set was downloaded from CPTAC data portal accession numbers PDC000272 and PDC000270 (file names for glycoproteomics: ′N-glycoproteomics_peptide_level_ratio_normal.txt` and ′N-glycoproteomics_peptide_level _rati_tumor.txt` and file name for protein normalization: `proteomics_gene_level_MD_abundance_normal.txt` and ′proteomics_gene_level_MD_ abundance_tumor.txt`), and the identified glycopeptides at each site were used for subsequent analysis. The relative glycosylation site occupation of TIMP-1 N30 and N78 glycosylation sites per patient were calculated as described previously (57). Briefly, the sum of all detected glycans at each of the two glycosylation sites (from the glycoproteome data set) was divided by the abundance of the TIMP-1 protein (from the proteome data set) for each patient.

### Purification of TIMP-1 from plasma

TIMP-1 from blood plasma was purified employing a four-step purification protocol on an ÄKTA pure system (ÄKTA pure 25 equipped with F9-C fraction collector and modules V9-Inj, V9-S, V9-C, U9-M, and C9, Cytiva Europe GmbH). Plasma was thawed on ice and diluted in IEX equilibration buffer (20 mM Hepes, pH 7.4, 5 mM EDTA, 0.05% Brij35) to a volume of 300 ml and then applied to an anion exchange chromatography column (Q-sepharose, Cytiva Europe GmbH). Bound proteins were eluded with IEX elution buffer (IEX equilibration buffer + 2M NaCl). In order to isolate TIMP-1 from the remaining proteins, TIMP-1–containing flow-through was applied on a pseudo-affinity chromatography (PAC) column (Blue-sepharose 6 fast flow, Cytiva Europe GmbH) and TIMP-1 was eluded with PAC elution buffer (20 mM Hepes, pH 7.4, 2M NaCl, 5 mM EDTA, 0.05% Brij35). For the depletion of antibodies, the PAC elution was applied to a ProteinG affinity column (Cytiva Europe GmbH). Flow-through of the ProteinG affinity column was then concentrated to 3-5 ml using Amicon Ultra 15 centrifugal filters (Merck KGaA), with a molecular cutoff of 10 kDa and applied on two size-exclusion chromatography columns wired in line (Hiload superdex 16/600 75pg coupled to a Hiload superdex 26/600 75pg, both Cytiva Europe GmbH). Fractionation of 2 ml fractions started from 0.25 column volumes to 1 column volume in 96-deep well plate (Eppendorf SE) and stored at −20 °C until analysis.

### Generation and purification of TIMP-1 glycosylation variants

Recombinant TIMP-1 glycosylation variants were created by mutation of serine or threonine in the glycosylation acceptor sites N-X-S/T (X every amino acid except proline) to an alanine (N-X-A) or valine (N-X-V) in the TIMP-1 coding sequence, using the Gibson assembly approach. TIMP-1 fragments harboring the necessary mutations were created in PCR reactions using the Phusion High-Fidelity PCR Kit (New England Biolabs). PCR fragments were purified using the mi-PCR purification kit (Metabion), concentration was determined at A_260nm_ with a NanoDropOne (PeqLab), and fragments were assembled in the pcDNA3.4 expression vector (Thermo Fisher Scientific) employing the Gibson assembly Master Mix Kit (New England Biolabs) based on the manufacturer’s instructions. Assembled plasmids were amplified in *Escherichia coli*, purified using the NucleoBond Xtra Midi Kit für Plasmid DNA (Machery & Nagel), and correct TIMP-1 coding sequence was validated using Sanger Sequencing (Eurofins). Subsequently, TIMP-1–containing plasmids were transfected into HEK 293 Freestyle cells (Thermo Fisher Scientific) to create stable TIMP-1 variant-expressing cells. HEK 293 Freestyle cells were cultured in rotating disposable flasks at 37 °C, 8% CO_2_ to collect supernatant for TIMP-1 purification. Two liters of TIMP-1 variant supernatant was used for recombinant TIMP-1 purification as described previously (22, 31). After the purification of TIMP-1, TIMP-1–containing fractions were pooled, and purity and correct glycosylation were analyzed as previously described (22, 31).

### Immunoblot analysis of TIMP-1 protein

TIMP-1 protein was analyzed using western blot as previously described (22). Briefly, TIMP-1–containing samples were mixed in loading buffer (0.2 M Tris, 2% SDS, 10% glycerol, 0.01% bromphenol blue, 0.05 M DTT) and incubated for 10 min at 95 °C. Samples were centrifuged (13,000*g*, 1 min) and separated by a 15% SDS PAGE. Proteins were transferred onto a nitrocellulose membrane (Amersham Bioscience) using a TransBlot semidry blotting system (Bio-Rad Laboratories Inc). Subsequently, membranes were blocked with 5% bovine serum albumin (BSA) in TBS-T (1 × Tris buffered saline (20 mM Tris/HCl pH 7.4 with 150 mM NaCl) containing 0.1% Tween 20) for 1h at RT and incubated with primary antibodies against TIMP-1 (1:1000; rabbit anti-human antibody; cat. #8946, Cell Signaling Technology) diluted in 5% BSA in TBS-T overnight at 4 °C. Membranes were washed three times with TBS-T at RT and incubated with secondary goat anti-rabbit IgG HRP-conjugated antibody solutions (1:5000; cat. #31462; Thermo Fisher Scientific Inc) for 1h at RT. Membranes were washed three times with TBS-T and bands were visualized using the Pierce ECL substrate (Thermo Fisher Scientific Inc), imaged with ChemiDoc imaging system, and quantified with the ImageLab Software (both from BioRad Laboratories Inc).

### Characterization of TIMP-1 glycosylation *ex vivo*

For the characterization of TIMP-1, *ex vivo* SEC fractions that contain only one of the three variants were used. Concentration of TIMP-1 in these fractions was analyzed by bicinchoninic acid protein assay (Thermo Fisher Scientific Inc), and 100 ng of total protein were used for analyses. For sequential deglycosylation analysis, TIMP-1–containing fractions were treated with one unit of PNGase F (cat#: P7367, Sigma-Aldrich) for 0, 30, and 60 min according to the manufacturer protocol and afterward analyzed *via* western blot. To determine site-specific glycosylation, TIMP-1 variants were incubated for 4h with 5U of neutrophil elastase (cat#: 324681, Sigma-Aldrich), subsequently digested with PNGase F based on the manufacturer protocol, and analyzed using Western blot.

### Analysis of published single-cell sequencing data set

For the analysis of OST complex expression, a publicly available scRNA data set was used. Raw data of scRNA-seq from pancreatic tumors and normal adjacent pancreas tissue of PC patients were obtained from the GEO database (GSE1556986). Filtered feature-barcode matrices of the dataset were then controlled for quality, processed, explored, and visualized using Trailmaker (https://app.trailmaker.parsebiosciences.com; Parse Biosciences, 2024) according to the software’s recommendations. Matrices were uploaded to Trailmaker and a set of quality filters applied to remove barcodes corresponding to the following categories: possible dying, dead, or stressed cells with high proportions of mitochondrial content setting a threshold on a per sample basis (threshold range: 7.77–50%); outliers in the distribution of number of genes *versus* number of transcripts by fitting a linear regression model (*p*-values between 0.07 and 0.000001); nuclei with high probability of being doublets using the scDblFinder method (threshold range: 37.3% to 88.8%). By integrating with Harmony (number of highly variable genes = 2000) and clustering with the Louvain method, we constructed a preliminary dataset, which was further corrected for one cluster with low numbers of genes per cell (Log_10_ number of genes <3.16) for downstream analyses. Cell types were annotated based on previous described marker genes (32, 33). For further analysis of cells from epithelial origin (identified by high expression of CTRB2, PRSS1, and REG1A for cells of acinar origin and KRT8, KRT18, and KRT19 for cells of ductal origin) were subclustered, and clusters were annotated as described above. For comparison of interested gene expression, normalized expression values of the respective clusters were exported using the normalized gene expression function of Trailmaker.

### Targeted RNA expression analysis of STT3A and STT3B

For targeted RNA expression analysis, total RNA from cells was isolated using TRIzol reagent (Thermo Fisher Scientific) as described previously (58). Reverse transcription for subsequent analysis of mRNA expression levels was performed using the High Capacity cDNA Reverse Transcription Kit (Applied Biosystems, Thermo Fisher Scientific) according to the manufacturer’s instructions. Primer sequences were designed using NCBI Primer Blast tool (59) (Primers: STT3A: forward: 5′-AACCCTGAGAGATATGGCTGG-3′, reverse: 5′-CAGACGA GTGGAGAAGGATAATAC-3′; STT3B: forward: 5′-GCAGGTGCTGTGTTCCTTAGTG-3′, reverse: 5′-GTCGTAGGTTGATGCTCAGACAC-3′). Real-time quantitative RT-PCR was performed by using Takyon No ROX SYBR 2× MasterMix blue dTTP (Eurogentech) on a Lightcycler480 System (Roche) according to the manufacturer’s instructions. 18S ribosomal RNA (Primers: forward: 5′-CCATCCAATCGGTAGTAGCG-3′, reverse: 5′-GTAACCCGTTGAACCCCATT-3′) was used as a reference gene for normalization.

### Analysis of TIMP-1 in cell culture supernatants

To analyze secreted TIMP-1 in the supernatant of pancreatic cells, 0.5 × 10^4^ cells/well were seeded in triplicate in 24-well plates. Twenty four hours after seeding, cells were washed twice with 1× PBS and cultured in DMEM with 5% pannexin CD (Pan Biotech GmbH) serum replacement for 48h, and the supernatant collected. Afterward, secreted TIMP-1 was analyzed using western blot as described above.

### Treatment of PC cell lines with NGI-1

To analyze the effects of OST complex inhibition on TIMP-1 glycosylation, 0.5 x 10^4^ cells/well were seeded in triplicates in 24-well plates. Twenty four hours after seeding, cells were washed twice with 1× PBS and cultured in DMEM with 5% pannexin CD (Pan Biotech GmbH) serum replacement in the presence of 1, 5, or 20 μM NGI-1 (cat#: SML1620 Sigma-Aldrich, dissolved in DMSO) or DMSO for 48 h, and the supernatant collected. Afterward, secreted TIMP-1 was analyzed using western blot as described above.

### TIMP-1 knockout in PC cells

For the generation of a TIMP-1 knockout in MIAPaCa-2 and T3M4 cell lines, the MISSION CRISPR plasmid system (Sigma Aldrich) was employed. Each CRISPR plasmid encoded for one gRNA, Cas9 protein, and a fluorescent protein (either GFP or RFP) for positive selection.

For knockout generation in PC cells, 2.5 x 10^6^ MIAPaCa-2 or T3M4 cells were seeded in two 10 cm dishes (TPP). On the next day, cells were transfected with 5 μg of each CRISPR plasmid (gRNA for the GFP plasmid: 5′-TAGACGAACCGGATGTCAG-3′; gRNA for the RFP plasmid: 5′-AAACTCCTCGCT GCGGTTG-3′) using lipofectamine 2000 (Thermo Fisher Scientific) after the manufacturer’s protocol. After 48 h, cells were washed with PBS, detached from plates by incubation in Accutase solution (Sigma-Aldrich) for 5 min, resuspended in FACS buffer (PBS with 1% FCS and 2 mM EDTA), and sorted for GFP and RFP expression on a SH800S cell (Sony biotechnology) equipped with a 100 μM sorting chip. After the exclusion of doublets, cells were gated for GFP and RFP positive staining, and highly double-positive cells were sorted into pure FBS. Sorted cells were counted manually using a Neubauer chamber, and single cell dilutions (3 cells per mL) were prepared. Hundred microliters per well of these dilutions were seeded in 96-well plates (TPP) and checked regularly for growing clones. Successful knockout is generated by the cleavage of Cas9 at both gRNA positions, leading to the deletion of 64 bp (Fig. S3*D*). This is designed to induce a frameshift in the TIMP-1 sequence after the amino acid A56 and introducing a premature stop codon (G68 mutated to stop), which results in a putative truncated TIMP-1–related protein consisting of 86 amino acids. For the evaluation of TIMP-1 knockout in these clones, genomic DNA was extracted using the DNA QuickExtract solution (LCG Biosearch Technologies) based on the manufacturer's instructions. The Phusion High Fidelity PCR kit (NEB) was used to amplify the targeted region of the genomic TIMP-1 sequence (fw:5′-TGGCTCATGCAGTCCATTTGACTC-3′; rev: 5′-CGACAAGCAGCTTGTGATTGGC-3′), which was analyzed using a 1.5% agarose gel, and selected clones were sequenced. Therefore, PCR product of selected clones was purified using the miPCR purification kit (Metabion) and sequenced by Sanger sequencing (Eurofins Genomics, primer:5`-TCCTCTCCTGCAGTCATCAG-3′). For the evaluation of TIMP-1 knockout on the protein level, clones were seeded in 24-well plates in complete media. Twenty four hours after seeding, cells were washed twice with 1× PBS and cultured in DMEM with 5% pannexin CD (Pan Biotech GmbH) serum replacement for 48 h. Afterward, secreted TIMP-1 in the collected supernatants was analyzed using western blot.

### Lentiviral-based overexpression of TIMP-1 glycosylation variants in PC cell lines

For the overexpression of the TIMP-1 glycosylation variants in PC cell lines, the pHIV-7–based vector system was employed as previously described (58). Lentiviral particles were produced in HEK293T, and supernatants containing lentiviral particles that either overexpress RFP as protein-overexpressing control or the four different TIMP-1 glycosylation variants were used to transduce PC cell lines with ablated endogenous TIMP-1. Successful overexpression was analyzed with an anti-TIMP-1 western blot.

### Proliferation assay of PC cells

Proliferation of the human PC cells was analyzed using the eBioscience cell proliferation dye eFluor450. For the analysis of cell proliferation, cells were stained using the eBioscience cell proliferation dye eFluor450 (Thermo Fisher Scientific) after the manufacturer’s protocol. Then 3 x 10^4^ cells per well were seeded in 48-well plates and cultured in DMEM including 10% FBS for 48h at 37 °C, 5% CO2. For flow cytometric analysis, cells were washed with PBS, detached from plates by incubation in Accutase solution (Sigma-Aldrich) for 5 min, resuspended in FACS buffer (PBS with 1% FCS and 2 mM EDTA), and transferred into v-bottom plates (TPP Techno Plastic Products AG). After centrifugation for 4 min at 650*g* and 4 °C, cell pellets were washed with flow buffer (10 mM Hepes/NaOH, pH7. 4, 140 mM NaCl, 2.5 mM CaCl_2_) and centrifuged again. Next, cells were resuspended in flow buffer, stained with 7-AAD (1:20 diluted; cat#: 420403; Biolegend) according to the manufacturer’s instructions, and filtered through a 41 μm polyamide mesh (NeoLab). Flow cytometry was performed as previously described (31). For the analysis of proliferation, cells were gated for 7-AAD negative staining after exclusion of cell debris and doublet cells and the geometric mean of eFluor450 staining was used for calculation of the fold change in proliferation between the conditions.

### Survival assay of PC cells

Survival of TIMP-1 glycovariant-overexpressing PC cells was analyzed using 7-AAD staining. 3 × 10^4^ cells per well were seeded in 48-well plates and cultured in DMEM including 10% FBS for 48h at 37 °C, 5% CO2. For flow cytometric analysis, cells were stained and processed as above. Flow cytometry was performed as previously described (31). After excluding cell debris and doublet cells, cells were gated for 7-AAD positive staining and the percentage of 7-AAD was used for the calculation.

### Lentiviral-based knockdown of CD63 in PC cell lines

The lentiviral-based mission guide shRNA system (Sigma) was employed to knock down CD63 in PC cell lines. Lentiviral particles containing a nontargeted control shRNA or one of two shRNAs against CD63 (5′- GCTGGCTATGTGTTTAGAGAT-3′or 5′- GCAAGGAGAACTATTGTCTTA-3′) were produced in HEK293T cells, and knockdown in the PC cells was performed as previously described (58).

### Flow cytometry–based TIMP-1/CD63–binding assay

We modified a published protocol (60) for analyzing ligand/receptor interactions *via* flow cytometry to analyze the impact of glycosylation site occupation on the interaction of TIMP-1 with the tetraspanin CD63. TIMP-1 KO T3M4 cells were detached with Accutase solution (Sigma-Aldrich), and 1 × 10^5^ cells per well were seeded in a 96-well V-bottom plate. Cells were washed two times with cold PBS (650*g*, 4 min, 4 °C) and incubated with equimolar doses of recombinant TIMP-1 in 1% BSA in PBS for 10 min on ice. Subsequently, cells were washed two times with ice-cold PBS (650*g*, 4 min, 4 °C), fixed with 2% PFA for 10 min on ice, and washed again two times with ice-cold PBS (650*g*, 4 min, 4 °C). Then, cells were stained for TIMP-1 (primary: anti-TIMP-1 rabbit mab (1:50; cat#: cell signal) and secondary: anti-rabbit donkey ab (1:5000; cat#: A48258, Thermo Fisher Scientific)) and CD63 (1:100; anti-human CD63-PerCP-Cy5.5; cat#: 353020, Biolegend) as described before (22, 31) and analyzed on the ID7000 flow cytometer (Sony). For the analysis of TIMP-1 binding to CD63, single cells were gated from all cells, the geometric mean of TIMP-1 staining of CD63-positive single cells was extracted, and TIMP-1 staining of the individual TIMP-1 glycosylation variants was normalized to background staining by subtraction from untreated control.

### MMP activity assays

For all performed assays, full-length recombinant human MMPs produced in eukaryotic cell lines were purchased from commercial providers (Biotechne: MMP-3 (cat#: 513-MP), Biolegend: MMP-1 (cat#: 592904), MMP-2 (cat#: 554304), MMP-7 (cat#: 761304), MMP-8 (cat#: 556104), MMP-9 (cat#: 755204)). MMP activity assays were performed as described previously (31). MMPs were activated based on the manufacturer’s instructions. Briefly, MMP-3 was activated with four-fold lower concentrations of chymotrypsin (cat#: C-3142, Sigma-Aldrich) for 30 min at 37 °C and the activation was stopped with 2 mM PMSF (cat#: P-7626, Sigma-Aldrich). All other MMPs were activated with p-aminophenylmercuric acetate (APMA, cat#: A9568, Sigma-Aldrich) at 37 °C (MMP-1, 1 mM APMA for 2h; MMP-2, 1 mM APMA for 1h; MMP-7, 1 mM APMA for 1h; MMP-8, 1 mM APMA for 1h; and MMP-9, 1 mM for 24h). After activation, MMPs were incubated with a molarity range of recombinant TIMP-1 variants for 30 min at 37 °C (MMP-1: 5 nM- 4.88 pM; MMP-2: 20 nM- 39.1 pM; MMP-3: 3 nM- 15.6 pM; MMP-7: 50 nM- 19.5 pM; MMP-8: 10 nM- 0.17 nM; and MMP-9: 160 nM-0.3125 nM) in a black 96-well plate (Thermofisher Scientific), and quenched fluorogenic substrate (FS-6 or NFF-3, both from Biotechne) was added. Increase of fluorescence over time was measured in a Spark M10 multi-plate reader (TECAN) at 393 nm. From these fluorescence *versus* time curves, the initial reaction velocities were retrieved and normalized to the initial velocity of the noninhibited MMP as the maximum, and the fluorescence decrease in the substrate without any TIMP-1 or MMP as the minimum. The normalized initial velocities were plotted against the recombinant TIMP-1 glycosylation variant inhibitor concentration in GraphPad Prism v10. For calculation of the TIMP-1 glycosylation-dependent MMP inhibitory constants K_i_ and IC_50_ values, the GraphPad Prism–integrated Morrison equation or activity *versus* inhibitor concentration curve fitting models were employed. For the calculation, at least three independent biological replicates per MMP and TIMP-1 glycosylation variant were used. The significance between the K_i_ and IC_50_ values for one MMP was determined based on the best-fit values.

### Statistical analysis

Statistical analysis was performed using GraphPad Prism (version 9.3.1, Graphpad Software Inc). Normal distribution was assessed using the Shapiro–Wilk normality and Kolmogorov–Smirnov normality test. Two independent groups were compared employing the Student’s *t* test for independent samples in case of normal distribution or employing the nonparametric Mann-Whitney test for independent samples in the absence of normal distribution. Statistical differences between groups were assessed with a one-way ANOVA Dunnett test for multiple comparisons between each group and the control. The significance of MMP activity data (K_i_ and IC_50_ values) was determined using the extra sum-of-squares F test based on the best-fit values of at least three independent biological replicates using nonlinear regression with Morrison K_i_ or inhibitor concentration *versus* response models for K_i_ or IC_50_ calculation (61), respectively. Outliers (Q = 1%) were detected and eliminated (62). Time-dependent overall survival probabilities were estimated with the Kaplan–Meier method. Groups separation was determined using the median as cutoff for patients with high abundance (HI, abundance above median) and low abundance (LO, abundance below median) (63). The log-rank test (Mantel-Cox test) was used to compare statistically significant differences between independent subgroups, as previously described (64).

## Data availability

Further information and requests for resources and reagents should be directed to and will be fulfilled by the corresponding author Achim Krüger (achim.krueger@tum.de) upon reasonable request and if approved by the Ethics Committee of the Medical Faculty of the Technical University of Munich.

## Supporting information

This article contains supporting information.

## Conflict of interest

The authors declare that they have no conflicts of interests with the contents of this article.
