# Supplementary material for: Extent of N-glycosylation of the metalloproteinase inhibitor and cytokine TIMP-1 determines pancreatic cancer cell proliferation and survival via CD63
Source: J Biol Chem. 2025 May 8;301(6):110211. doi: 10.1016/j.jbc.2025.110211 (PMC12167790; doi:10.1016/j.jbc.2025.110211)
Supplement: Supplemental Figure 1 [file mmc1.pdf]

Supplemental Figure 1: TIMP-1 glycosylation macroheterogeneity is altered in the plasma of PC patients.

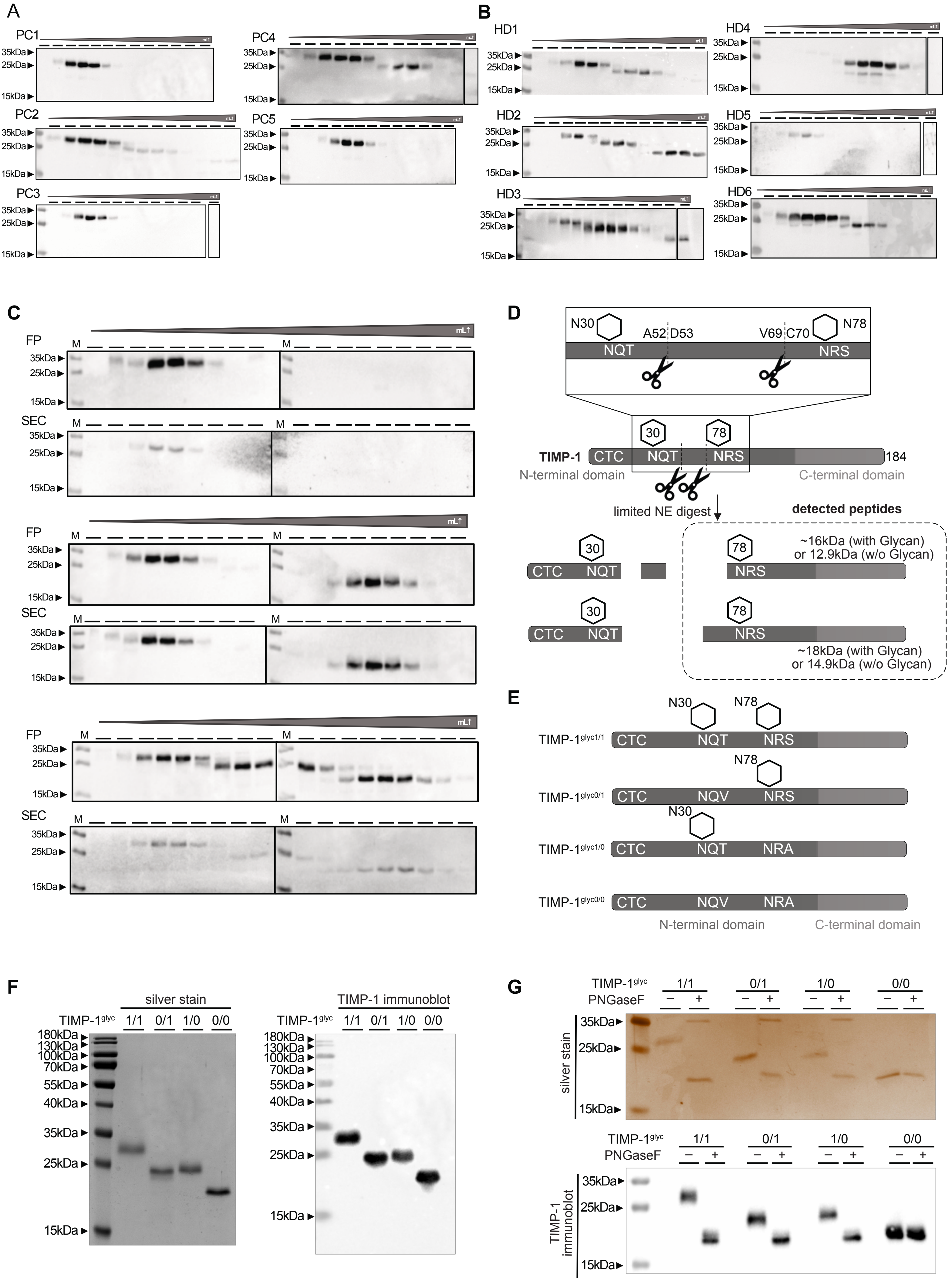

Supplemental Figure 1: TIMP-1 glycosylation macroheterogeneity is altered in the plasma of PC patients. **A, B**. Western blots of plasma TIMP-1 purified from pancreatic cancer patients (PC) (A) and healthy individuals (HD) (B) collected after size-exclusion chromatography (SEC). Representative samples for both PC (Fig. 1B) and HD (Fig. 1C) are included as PC1 and PC2 (A) and HD1 and HD2 (B), respectively, to allow comparison across all samples. **C**. TIMP-1 western blots of plasma TIMP-1 after SEC from three donors either purified with the full four column purification protocol (FP) or only SEC (SEC). **D**. Schematic depiction of neutrophil elastase (NE) cut sites in TIMP-1. NE is able to cut the TIMP-1 protein between the N30 (NQT) and N78 (NRS) glycosylation sites after the amino acids A52 and V69 (65). Therefore, limited NE digestion leads to the creation of five different peptides. Of these, the employed antibody interacting with the C-terminus, binding to the region around A107, detects the N78 harboring fragments with different molecular weights dependent on the presence or absence of the glycan. NE is represented as scissors; glycans are represented as hexagons. CTC are the first 3 amino acids of the mature secreted protein. **E**. Schematic depiction of the sequence of recombinant TIMP-1 glycosylation variants. Glycans are represented as hexagon **F**. Silver stain and TIMP-1 western blot of recombinant TIMP-1 glycosylation variant stocks. **G**. Silver stain and TIMP-1 western blot of recombinant TIMP-1 glycosylation variant stocks treated with or without PNGase F.
