# Supplementary material for: Extent of N-glycosylation of the metalloproteinase inhibitor and cytokine TIMP-1 determines pancreatic cancer cell proliferation and survival via CD63
Source: J Biol Chem. 2025 May 8;301(6):110211. doi: 10.1016/j.jbc.2025.110211 (PMC12167790; doi:10.1016/j.jbc.2025.110211)
Supplement: Supplemental Figure 2 [file mmc2.pdf]

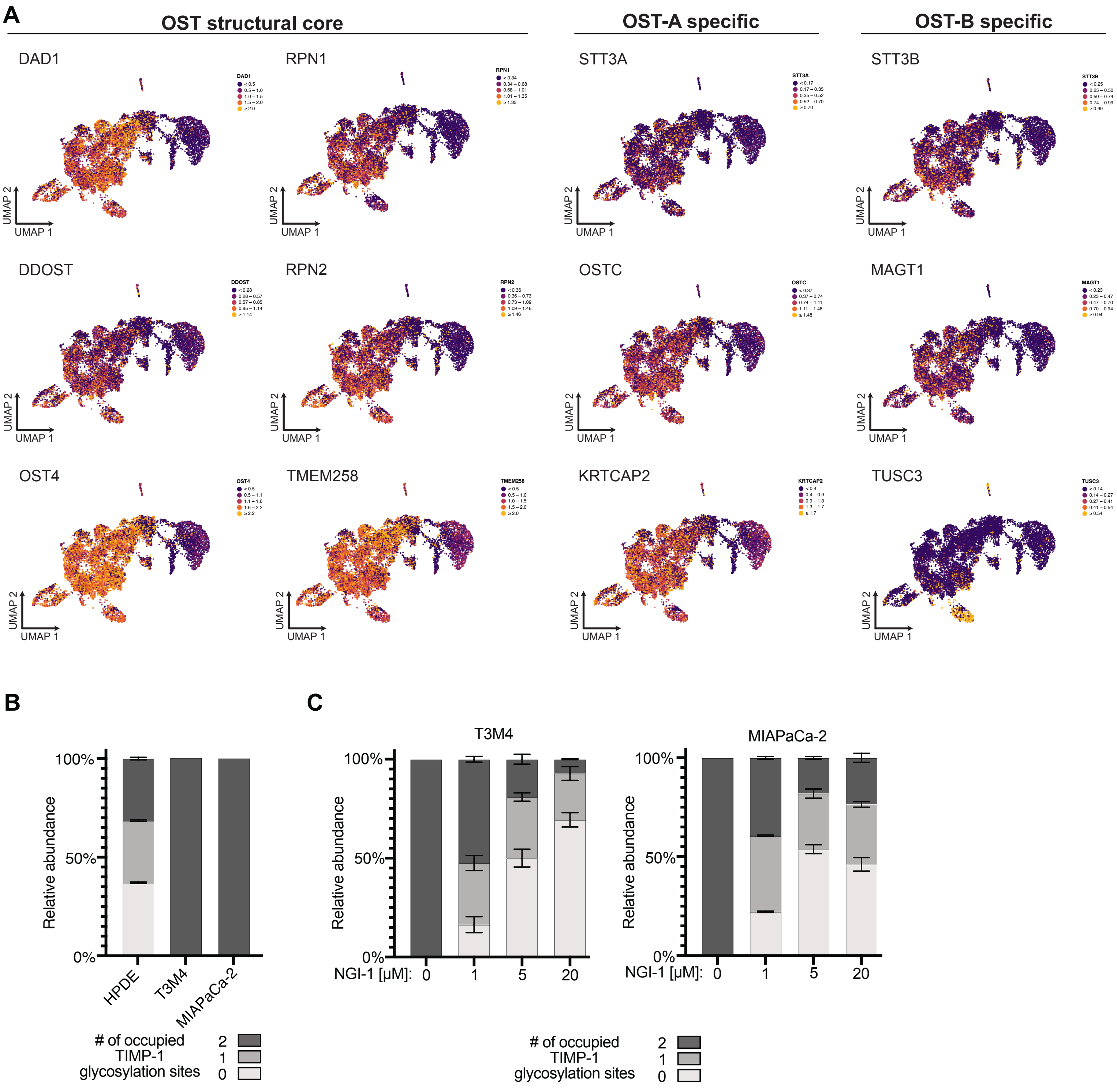
A
OST structural core
OST-A specific
OST-B specific
DAD1
RPN1
STT3A
STT3B
DDOST
RPN2
OSTC
MAGT1
OST4
TMEM258
KRTCAP2
TUSC3
UMAP 1
UMAP 2
B
C
T3M4
MIAPaCa-2
Relative abundance
100%
50%
0%
HPDE
T3M4
MIAPaCa-2
NGI-1 [μM]: 0 1 5 20
# of occupied TIMP-1 glycosylation sites
2
1
0

**Supplemental Figure 2:** Single-cell RNAseq analysis reveals an upregulation of the Oligosaccharyltransferase (OST) complex in pancreatic cancer cells. **A**. UMAP of the expression of the OST complex members in the sub-clustered non-cancerous epithelial cells of adjacent/normal pancreas (n=3) and PC tissues (n=16). The color of each dot represents the expression value of the OST complex member in the respective cell (see legend). **B**. Quantification of the TIMP-1 western blots of the supernatant of non-tumorous pancreatic HPDE cells and pancreatic tumor cell lines MIAPaCa-2 and T3M4. **C**. Quantification of the TIMP-1 western blots of the supernatant of pancreatic cancer cell lines MIAPaCa-2 and T3M4 which were treated with 0, 1, 5, and 20 μM NGI-1 for 48h.
