# Supplementary material for: Extent of N-glycosylation of the metalloproteinase inhibitor and cytokine TIMP-1 determines pancreatic cancer cell proliferation and survival via CD63
Source: J Biol Chem. 2025 May 8;301(6):110211. doi: 10.1016/j.jbc.2025.110211 (PMC12167790; doi:10.1016/j.jbc.2025.110211)
Supplement: Supplemental Figure 3 [file mmc3.pdf]

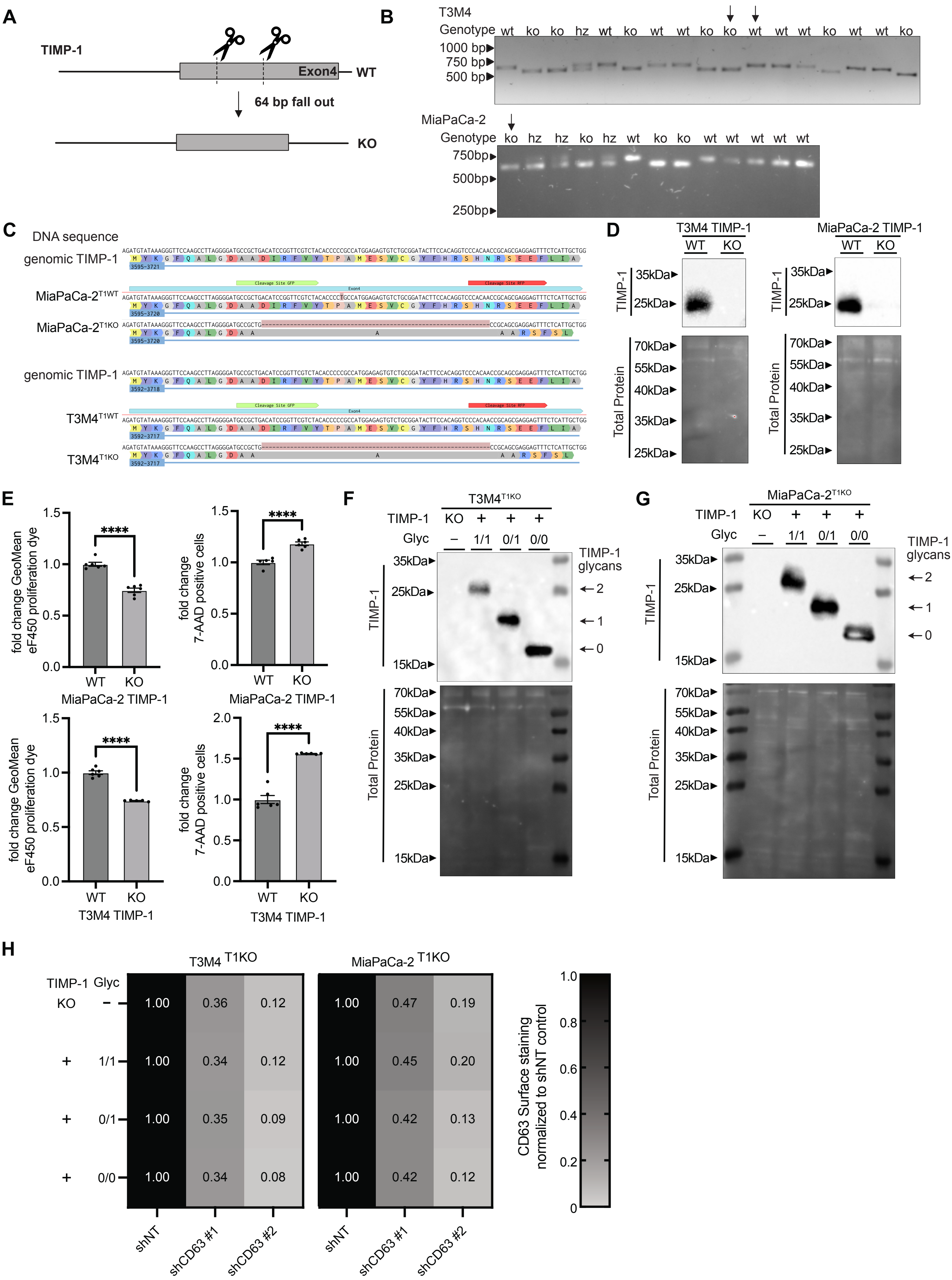
A
TIMP-1
Exon4
WT
64 bp fall out
KO
B
T3M4
Genotype wt ko ko hz wt ko wt wt ko ko wt wt wt ko wt wt ko
1000 bp
750 bp
500 bp
MiaPaCa-2
Genotype ko hz hz ko hz wt ko ko wt wt wt wt wt
750bp
500bp
250bp
C
DNA sequence
genomic TIMP-1
MiaPaCa-2T1WT
MiaPaCa-2T1KO
genomic TIMP-1
T3M4T1WT
T3M4T1KO
D
T3M4 TIMP-1
WT KO
MiaPaCa-2 TIMP-1
WT KO
TIMP-1
35kDa
25kDa
Total Protein
70kDa
55kDa
40kDa
35kDa
25kDa
E
fold change GeoMean eF450 proliferation dye
fold change 7-AAD positive cells
WT KO
MiaPaCa-2 TIMP-1
T3M4 TIMP-1
****
F
T3M4T1KO
TIMP-1 KO + + +
Glyc – 1/1 0/1 0/0
TIMP-1 glycans
← 2
← 1
← 0
35kDa
25kDa
15kDa
70kDa
55kDa
40kDa
35kDa
25kDa
15kDa
G
MiaPaCa-2T1KO
TIMP-1 KO + + +
Glyc – 1/1 0/1 0/0
TIMP-1 glycans
H
T3M4 T1KO
MiaPaCa-2 T1KO
TIMP-1 Glyc
KO – 1.00 0.36 0.12 1.00 0.47 0.19
+ 1/1 1.00 0.34 0.12 1.00 0.45 0.20
+ 0/1 1.00 0.35 0.09 1.00 0.42 0.13
+ 0/0 1.00 0.34 0.08 1.00 0.42 0.12
shNT shCD63 #1 shCD63 #2
CD63 Surface staining normalized to shNT control
1.0 0.8 0.6 0.4 0.2 0

Supplemental Figure 3: Flow cytometric analysis reveals TIMP-1 glycosylation/CD63-dependent induction of tumor cell proliferation and survival. **A-D**. Generation and validation of TIMP-1 knockout in pancreatic cancer cells. **A**. TIMP-1 knockout was generated using a two-gRNA approach targeting exon 4. Targeting of both gRNAs leads to a deletion of a 64bp fragment, triggering a frameshift and premature stop codon in the TIMP-1 protein. **B**. Validation of genomic TIMP-1 knockout single-cell clones of T3M4 and MIAPaCa-2 cells. A PCR fragment of 628 bp is present in cells with a wild-type TIMP-1 allele. A PCR fragment of 564bp is present in cells with a deleted TIMP-1 allele. wt: only wildtype alleles; hz: wildtype and deleted alleles; ko: deleted alleles. Arrows indicate the used clones. **C**. Sequencing results of the TIMP-1 wild-type and knockout T3M4 and MIAPaCa-2 cells covering exon 4 of TIMP-1. **D**. TIMP-1 western blot of supernatant from the TIMP-1 wild-type and selected knockout T3M4 and MIAPaCa-2 cell lines. **E**. Quantification of proliferation and cell death of wildtype and TIMP-1 knockout cell lines by flow cytometry. MIAPaCa-2^{T1WT}, MIAPaCa-2^{T1KO}, $T3M4^{T1WT}$, and $T3M4^{T1KO}$ cells were stained with an eFluor450 proliferation dye and cultivated for 48h. Proliferation was evaluated by calculation of the geometric means of eFluor450 staining. The background of non-eFluor450 stained cells was subtracted, and the fold change of the reciprocal eFluor450 geometric means was calculated by normalization to control cells. Data represents n=6 biological replicates each experiment before outlier identification. MIAPaCa-2^{T1WT}, MIAPaCa-2^{T1KO}, $T3M4^{T1WT}$, and $T3M4^{T1KO}$ cells were cultivated for 48h and amount of death cells were analyzed by positive staining for 7-AAD. Fold change of percentages were calculated by normalization to control cells. Data represents n=6 biological replicates for each experiment. Quantification of proliferation by analyzed by flow cytometry. **F**. TIMP-1 western blot and total protein stained with SYPRO Ruby total protein blot stain of supernatant from $T3M4^{T1KO}$ cells transduced with virus leading to the expression of TIMP-$1^{glyc1/1}$, TIMP-$1^{glyc0/1}$, or TIMP-$1^{glyc0/0}$, or mCherry (control cells). **G**. TIMP-1 western blot and total protein stained with SYPRO Ruby total protein blot stain of supernatant from MIAPaCa-2^{T1KO} cells transduced with virus leading to the expression of TIMP-$1^{glyc1/1}$, TIMP-$1^{glyc0/1}$, or

TIMP-1$^{glyc0/0}$, or mCherry (control cells). **H**. Validation of CD63 knockdown in T3M4^{T1KO} and MIAPaCa-2^{T1KO} cells expressing TIMP-1$^{glyc1/1}$, TIMP-1$^{glyc1/1}$, or TIMP-1$^{glyc0/0}$, or control cells. Fold change of surface CD63 staining was evaluated using the geometric mean of CD63-PE staining and normalization to non-targeted control cells. TIMP-1$^{glyc1/1}$ harbors two glycans at N30 and N78, TIMP-1$^{glyc0/1}$ harbors one glycan at N78 and no glycan N30, and TIMP-1$^{glyc0/0}$ harbors no glycans at N30 and N78. Statistical analysis was performed by Student's t-tests or Mann-Whitney tests (E). Mean ± SEM (E) *, $p \leq 0.05$; **, $p \leq 0.01$; ***, $p \leq 0.001$; ****, $p \leq 0.0001$.
